# In Vitro Chondrogenesis Induction by Short Peptides of the Carboxy-Terminal Domain of Transforming Growth Factor β1

**DOI:** 10.3390/biomedicines11123182

**Published:** 2023-11-29

**Authors:** Maria Pitou, Eleni Papachristou, Dimitrios Bratsios, Georgia-Maria Kefala, Anastasia S. Tsagkarakou, Demetrios D. Leonidas, Amalia Aggeli, Georgios E. Papadopoulos, Rigini M. Papi, Theodora Choli-Papadopoulou

**Affiliations:** 1 Laboratory of Biochemistry, School of Chemistry, Aristotle University of Thessaloniki (AUTh), 54124 Thessaloniki, Greece; 2 Laboratory of Biomedical Engineering, School of Chemical Engineering, Aristotle University of Thessaloniki (AUTh), 54124 Thessaloniki, Greece; 3 Department of Biochemistry and Biotechnology, University of Thessaly, Biopolis, 41500 Larissa, Greece

**Keywords:** transforming growth fβ1, cartilage repair, chondrogenesis, Smad signaling

## Abstract

Τransforming growth factor β1 (TGF-β1) comprises a key regulator protein in many cellular processes, including in vivo chondrogenesis. The treatment of human dental pulp stem cells, separately, with Leu^83^-Ser^112^ (C-terminal domain of TGF-β1), as well as two very short peptides, namely, 90-YYVGRKPK-97 (peptide 8) and 91-YVGRKP-96 (peptide 6) remarkably enhanced the chondrogenic differentiation capacity in comparison to their full-length mature TGF-β1 counterpart either in monolayer cultures or 3D scaffolds. In 3D scaffolds, the reduction of the elastic modulus and viscous modulus verified the production of different amounts and types of ECM components. Molecular dynamics simulations suggested a mode of the peptides’ binding to the receptor complex TβRII-ALK5 and provided a possible structural explanation for their role in inducing chondrogenesis, along with endogenous TGF-β1. Further experiments clearly verified the aforementioned hypothesis, indicating the signal transduction pathway and the involvement of TβRII-ALK5 receptor complex. Real-time PCR experiments and Western blot analysis showed that peptides favor the ERK1/2 and Smad2 pathways, leading to an articular, extracellular matrix formation, while TGF-β1 also favors the Smad1/5/8 pathway which leads to the expression of the metalloproteinases ADAMTS-5 and MMP13 and, therefore, to a hypertrophic chondrocyte phenotype. Taken together, the two short peptides, and, mainly, peptide 8, could be delivered with a scaffold to induce in vivo chondrogenesis in damaged articular cartilage, constituting, thus, an alternative therapeutic approach for osteoarthritis.

## 1. Introduction

Osteoarthritis (OA) is the most prevalent, degenerative joint disease that slowly leads to disability. OA mainly affects knee joints and, secondly, other joints including those of the upper and lower limbs, spine, and hips [1]. Its etiopathogenesis and pathophysiology have not been clearly elucidated, but the main facts include articular cartilage degradation, subchondral bone changes, and induction of inflammation [2]. The gradual degeneration of the articular matrix is characterized by the breakdown of the two basic components of the extracellular matrix, i.e., collagen type II and aggrecan [3,4], while bone remodelling is characterized by osteophyte formation [5].

Currently, there is no cure available for OA, only drugs and assistive methods which ease pain and delay the process of cartilage degeneration [5]. Many novel therapeutic approaches focus on cartilage regeneration and in situ chondrogenic differentiation of human mesenchymal stem cells (hMSCs), while others focus on in vitro chondrogenic differentiation and transplantation [6,7,8]. According to studies, human dental pulp stem cells (hDPSCs) show a chondrogenic capacity and are of great interest for cartilage repair because of their easy isolation and expansion potential [9,10].

Previous studies have shown that cartilage maintenance and regeneration is a complex procedure controlled by different regulatory factors and pathways, involving TGF-β, BMP, and Wnt signalling. Members of the TGF-β superfamily play an important role in cartilage maintenance and ΤGF-β1, especially, exhibits an anabolic role in articular cartilage [7,11]. Transforming growth factor β1 (ΤGF-β1) is a multifunctional cytokine that belongs to the TGF-β superfamily. It possesses a crucial role in extracellular matrix formation, cell growth, cell differentiation, and apoptosis [12,13]. It is normally expressed in many tissues and is the predominant isoform in articular cartilage [14].

In healthy joints, TGF-β1 is stored extracellularly as an inactive form, and, when needed, it matures and promotes chondrocyte proliferation and the differentiation of chondroprogenitors to chondroblasts [15]. Chondrogenesis and, consequently, extracellular matrix formation is induced by TGF-β1 binding to the heterodimerized receptor complex, type II receptor (TβRII), with type I receptor (Activin Like Kinase 5, ALK-5) and signaling through the canonical Smad 2/3 pathway and the non-canonical, ΤAK1-JNK/p38 pathway. Transforming growth factor β-activated kinase 1 (TAK-1) is a mitogen protein 3 activated kinase with a key role in inflammation [16]. OA is associated with a reduced expression of ALK-5 which favors the ALK-1/Smad 1/5/8 pathway, leading to hypertrophy with increased type X and MMP13 expression [17]. Reduced TGF-β signaling in articular chondrocytes leads to a progressive OA-like phenotype in mice with the upregulation of matrix metalloproteinase-13 (MMP13) and a disintegrin and metalloproteinase with thrombospondin domain (ADAMTS-5). These are key enzymes to the proteolytic degradation of aggrecan and high amounts are present in the OA joint. In addition, it has been reported that TGF-β1 is a potent inducer of collagenase 3 (MMP13), a collagen type II degrading enzyme in human fibroblasts, and hepatic cells [18].

The aim of this study is to evaluate the in vitro chondrogenic differentiation capacity of short peptides of the C-terminal domain of TGF-β1 that contain the critical amino acids for receptor interaction. Specifically, a sequence of the C-terminal domain of TGF-β1 and two shorter peptides of this region, an octapeptide and a hexapeptide, were studied against the induction of the in vitro chondrogenesis of human dental pulp stem cells (hDPSCs) cultured in a monolayer or three-dimensional (3D) scaffolds. Moreover, the extracellular matrix homeostasis and the signaling pathways that lead to chondrocyte differentiation were investigated.

## 2. Materials and Methods

### 2.1. Bacterial Strains, Eukaryotic Cell Line, Media, and Chemicals

*E. coli* strain TOP10F was used for recombinant plasmid production, while *E. coli* strain BL21(DE3) was used for protein expression. Plasmid vectors pAN5 (Avidity, Aurora, CO, USA) and pET-29c(+) (Novagen, Madison, WI, USA) were used for subcloning and protein expression, respectively. Bacterial media were purchased from DIFCO Laboratories (Detroit, MI, USA) and AppliChem (Darmstadt, Germany). Other chemicals were purchased from Sigma-Aldrich (St. Louis, MO, USA) and Gibco (USA) and were of analytical or reagent grade.

Human dental pulp stem cells (hDPSCs) were kindly provided by the Assistant Professor A. Bakopoulou from the School of Dentistry, Aristotle University of Thessaloniki. Sample collection was conducted according to the relevant guidelines and regulations and had been approved by the Institutional Review Board of the Aristotle University of Thessaloniki (Nr. 66/18-06-2018). An informed consent form was signed by all the donors. Eukaryotic cell culture media and chemicals were purchased from Βiosera (Cholet, France) and Thermo Fischer (Gibco, Waltham, MA, USA).

### 2.2. Cloning

The sequence L^83^-S^112^ of the C-terminal domain of TGF-β1 was amplified from the recombinant vector pET-29c(+) that expresses mature TGF-β1. Primer pairs, restriction enzymes, and plasmid vectors used for amplification and cloning are provided at Table 1.

PCR amplification was carried out in 50 μL reaction with Q5^®^ High Fidelity (New England BioLabs, Ipswich, MA, USA, M0491S) following the manufacturer’s protocol. The PCR reaction was performed in a mix containing DNA template 10–20 ng, 0.5 μM of each primer, polymerase Buffer 1X, Q5^®^ High Fidelity polymerase 1 U/L, 200 mM of each dNTP, and ddH_2_O up to 50 μL, and the conditions used for the reaction were as follows: (a) initial denaturation at 98 °C for 30 s, 1 cycle, (b) denaturation at 98 °C for 20 s, 25 cycles, (c) annealing at 60 °C for 15 s, (d) extension at 72 °C for 30 s, (e) final extension at 72 °C for 7 min, and (f) storage at 4 °C. PCR product was resolved by electrophoresis at 90 V for 30 min in 1.5% *w*/*v* agarose gel in 1× TAE buffer and visualized with UV light. The L^83^-S^112^ C-terminal TGF-β1 PCR product and pAN5 vector were double-digested with HindIII and XhoII (New England Biolabs, NEB, Ipswich, MA, USA, R0146S, R0104S) at 37 °C for 1 h. The digested PCR product and the plasmid vector were purified with Qiaquick Gel Extraction Kit (Qiagen, Germantown, MD, USA, 28706X4) and the procedure was performed following the manufacturer’s protocol. Ligation reaction was performed at 16 °C for 16 h using T4 DNA ligase (Takara Holdings Inc., Kyoto, Japan, 2011A). *E. coli* strains were transformed, and positive colonies were tested with double digestion.

### 2.3. Overexpression and Purification of C-Terminal TGF-β1

The recombinant pET-29c(+) plasmid with the L^83^-S^112^ C-terminal region of TFG-β1 was transferred into *E. coli* strain BL21 (DE3). Τransformed cells were grown at 37 °C in LB culture medium (25 μg/mL kanamycin) until the optical density at 600 nm reached 0.6. Protein expression was induced with 1 mM isopropyl-d-thiogalactoside (IPTG) (Sigma-Aldrich, MA, USA, D8418) for another 3 h at 37 °C. The cells were harvested, suspended to lysis buffer (20 mM Tris–HCl, pH 8.0, 150 mM NaCl, Triton-X 0.1% *v*/*v*), and lysed with sonication. Cell lysate was centrifuged at 15,000 rpm for 15 min at 4 °C, the supernatant was discarded, and the procedure was repeated for 3 cycles. The pellet was resuspended to lysis buffer (20 mM Tris–HCl, pH 7.5, 150 mM NaCl, 6 M Urea) for 16 h at 4 °C and, after centrifugation, the supernatant was collected.

The soluble protein was further purified using affinity chromatography (HisTrap™ FF crude, GE Healthcare, Chicago, IL, USA) applied on AKTA purifier (GE Healthcare). The protein solution was loaded at 0.2 mL/min on the column equilibrated in Buffer A (20 mM Tris-HCl, pH 7.5, 150 mM NaCl, 5 mM Imidazole). The column was then washed with the same buffer to remove unbound proteins. The target protein was eluted at 0.2 mL/min with Buffer B (20 mM Tris-HCL, pH 7.5, 150 mM NaCl, 250 mM Imidazole). Elution fractions exhibiting high protein purity were determined via SDS-PAGE analysis. The same protocol was also used for the purification of L^83^-S^112^ C-terminal domain of TGF-β1. The quality and purity of the protein samples were evaluated by 15% *v*/*v* SDS-PAGE in the presence of 2-mercaptoethanol (β-ME) (Sigma-Aldrich, MA, USA, 444203) using Coomassie Brilliant Blue R-250 staining (Sigma-Aldrich, USA, 1.12553). After purification, the protein was dialyzed using Amicon filters (Millipore, Burlington, MA, USA, UFC500308).

### 2.4. Peptide Synthesis

Molecular simulation experiments of TGF-β1 binding to its receptors revealed the critical C-terminal region for this interaction. It was found that, in the sequence, L^83^-S^112^ is necessary for successful binding to the TβRII receptor, and, more specifically, Val92, Gly95, and Arg94 are in adjacent positions. Further, two peptides that contain the critical amino acids for binding to the receptor were chemically synthesized by GeneCust (France). The sequences are listed below (Table 2).

### 2.5. Cell Culture and Differentiation

hDPSCs were kindly provided by Associate Professor A. Bakopoulou from the School of Dentistry, Aristotle University of Thessaloniki and were isolated from the third molars of young adult healthy donor using the enzymatic dissociation method [19]. Cell cultured at passage 4 were used for in vitro differentiation experiments. hDPSCs were treated with 10 ng/mL full-length TGF-β1 [20] or equimolar concentration of the different peptides (C-terminal region L^83^-S^112^: 2.7 ng/mL, peptide 8: 789.06 pg/mL, peptide 6: 562.5 pg/mL) For cell differentiation experiments, three different experimental groups were studied, monolayer culture, alginate beads, and gelatin type B–alginate gels. Two different types of media were used, alpha MEM and Stem Pro Chondrogenenesis medium (Invitrogen, Waltham, MA, USA, A1007101), and a protease inhibitor cocktail for cell culture (Millipore Sigma, Burlington, MA, USA) was added in each medium.

Monolayer culture

At about 80% of confluence, the adherent hDPSCs were trypsinized, counted, and suspended to alpha MEM. Cells were seeded in 12-well plates (for Alcian Blue staining) and 6-well plates (for RNA and protein isolation) at a numerical density of 20,000 cells/mL (2361 cells/cm^2^ and 2105 cells/cm^2^, respectively). Once attached, cells were treated with proteins or peptides. Medium changed every 2–3 days. Cell cultures were incubated in a humidified atmosphere of 5% CO_2_ in air at 37 °C.

Alginate beads

At about 80% of confluence, the adherent hDPSCs were trypsinized, counted, and suspended in sterile 1.2% *w*/*v* alginate solution (low viscosity, dissolved in 1× PBS, pH 7.4) at a concentration of 20,000 cells/mL. The cell-laden solution was dropped slowly from a 16-gauge needle into a solution of 102 mM CaCl_2_. Upon contact with CaCl_2_ solution, the alginate/cell suspension formed spherical beads. After 10 min, the solution was removed, and the beads were washed with PBS 1×. Beads of the same size were selected, and 3–4 beads/position were placed in a 12-position plate overlaid with culture medium. Cultures were treated with proteins and peptides, and medium changed every 2–3 days.

Gelatin type B–alginate gels

The 3D culture composed of 6% gelatin type B and 5% alginate. Initially, the solid materials were sterilized in ultraviolet light for 1 h. Then, alginate was dissolved in a suitable volume of 1X PBS at 60 °C, the type B gelatin was added, and solubilization continued at 40 °C. An appropriate amount of gel was transferred to 12-well plates (200 μL/well), followed by the addition of 100 mM CaCl_2_ solution and incubation at 37 °C for 1 h to promote polymerization. After one hour, the 100 mM CaCl_2_ solution was removed, and gels were washed twice with 1× PBS pH 7.4. At about 80% of confluence, the adherent hDPSCs were trypsinized, counted, and seeded to the gels at a concentration of 20,000 cells/mL. The next day, the nutrient medium was changed and TGF-β1 or peptides were added. Cultures were treated with proteins and peptides, and medium changed every 2–3 days.

### 2.6. RNA Extraction

RNA isolation from monolayer cultures

Total RNA was extracted from hDPSCs treated with TGF-β1 and peptides of the C-terminal region on days 5, 14, and 21 using the Nucleospin^®^ RNA isolation kit (Machery-Nagel, Düren, Germany, 740955.50). The concentration and the purity of RNA in each sample were determined by measuring the optical density at 260 nm and the ratio of 260/280 nm, respectively.

RNA isolation from 3D cultures

Isolation of total RNA from cells encapsulated in alginate beads was conducted at 5, 14, and 21 days of chondrogenic differentiation. Alginate beads were transferred to the recovery solution (55 mM Na_3_C_6_H_5_O_7_, 15 M NaCl, 25 mM Hepes pH 7.0) and left for 10 min at 4 °C. Centrifugation at 11,000× g for 2 min at 25 °C followed and the cell pellet was washed 3 times with 1× PBS pH 7.4 at 25 °C. Cell lysis and isolation of total RNA was performed with Macherey-Nagel NucleoSpin^®^ RNA isolation kit (Machery-Nagel, Germany, 740955.50) according to the manufacturer’s protocol. Concentration and purity of RNA in each sample were determined by measuring the optical density at 260 nm and the ratio of 260/280 nm, respectively.

### 2.7. cDNA Synthesis

First-strand cDNA synthesis was performed for each RNA sample with M-MuLV Reverse Transcriptase (Minotech, Crete, Greece, 801-1(10KU)) according to the manufacturer’s protocol. Each reaction was prepared with 0.5 ng/mL total RNA, 5 µΜ Oligo(dT)20, and 500 µΜ of each dNTP. The reaction steps included heating to 65 °C for 5 min, cooling on ice for 1 min, and addition of 200 U of reverse transcriptase (RT), 1× RT Buffer, and 5 µΜ DTT. The mixture was primarily incubated at 25 °C for 5 min so Oligo(dT)20 annealed, and, consequently, cDNA synthesis was performed at 37 °C for 1 h. At a final step, inactivation of enzyme was performed at 70 °C for 15 min.

### 2.8. Real-Time Polymerase Chain Reaction

Real-time q-PCR analysis was carried out to estimate the expression levels of *COL2A1, ACAN, ADAMTS-5, MMP13, COL1A1, COL10A1, RUNX2, SOX9,* and *ALP*. Real-time qPCR analysis was performed in StepOne™ Real-time PCR System (ThermoFisher Scientic, Waltham, MA, USA), using KAPA SYBR^®^ FAST qPCR Kit Master Mix (2×) (Sigma-Aldrich, USA, KK4602), according to the manufacturer’s protocol. Gene expression was normalized against the housekeeping genes *GUSB* and *GAPDH,* and the values were obtained using the 2^−ΔΔCt^ method [21]. The primer pairs used in the real-time qPCR analysis are shown in Table 3. For all reactions, a Fast program was selected, and the annealing temperature was set at 60 °C.

### 2.9. Alcian Blue Staining and Semi-Quantification

hDPSCs seeded at the desirable concentration were washed with 1× PBS pH 7.4 and fixed with 4% *v*/*v* formalin buffer (Sigma-Aldrich, MA, USA, HT501128-4L) for 30 min at room temperature. Formalin was removed and cells were washed with sterile distilled water and incubated with Alcian Blue 8G^®^ 1% *w*/*v* in 0.1 N HCl (Sigma-Aldrich, MA, USA, 33864-99-2) for 1 h at room temperature. Afterwards, cells were washed three times with 0.1 N HCl and pH was neutralized with water. The cells were observed under Nikon Eclipse TS-100 inverted optical microscope and photographs of the stained monolayers were taken at 100× and 400× magnification.

Semi-quantification was conducted upon removal of staining and incubation of cells with 8 M guanidine hydrochloride (Sigma-Aldrich, USA, 50-01-1) buffer in 10 mM Na_2_HPO_4_(pH 7.0) at 4 °C for 12 h. The optical density (OD) was measured at 600 nm with a microplate reader (BioTek Instruments, Inc., BioTek, Winooski, VT, USA).

### 2.10. Western Blot Analysis

Total protein was isolated from hDPSCs treated with TGF-β1 or the different peptides for 5, 14, and 21 days. Cells were washed with 1X PBS and were lysed on ice using RIPA lysis buffer (1 M tris-HCl pH = 7.5, 5 M NaCl, 0.5% *v*/*v* sodium deoxycholate, 10% NP-40), 10 μM aprotinin, and 10 μM leupeptin to yield total cellular protein extracts after centrifugation at 10,000× *g* for 10 min. After isolation, protein concentrations were quantified via Bradford protocol. An equal amount of protein from each sample (50 μg) was separated on 12% SDS-polyacrylamide gel by electrophoresis and then transferred onto a nitrocellulose membrane at 60 mA. After blocking in 5% nonfat dry milk in PBS, the membranes were incubated overnight at 4 °C with the primary antibodies diluted in the same blocking buffer. All antibodies, ERK, pERK, Smad2, pSmad2, Smad1/5/8, pSmad1/5/8, and GAPDH (Cell Signaling, Sigma, MA, USA) were monoclonal rabbit anti-human and used in the dilution 1:1000. GAPDH was used as a loading control. Following incubation with the antibodies, the membranes were then washed with PBS/0.05% Tween 20 and incubated with secondary goat anti-rabbit conjugated alkaline phosphatase (ALP) (Cell Signaling, Sigma, MA, USA) antibody (1:2000) for 1 h at room temperature. After the incubation, membranes were washed with PBS 1×/0.05% Tween 20 and developed with the addition of BCIP/NBT in ALP buffer.

### 2.11. Rheological Measurements

A stress-controlled AR-G2 rheometer by TA Instruments was employed, operating at 37.0 ± 0.1 °C. Three types of rheological measurements were carried out, in each case with a fresh sample to avoid any pre-treatment effect: dynamic oscillatory strain sweeps and dynamic oscillatory time sweeps in the linear viscoelastic regime (LVR), from which values of elastic (G′) and viscous (G″) moduli are obtained, and steady-state flow steps, from which values of dynamic viscosity values are a function of shear rate, are derived. In the time sweep measurements, the geometry was oscillating with a frequency of 3.14 rad/s (0.5 Hz) 6.28 rad/s (1 Hz), with a duration of 10 min; the initial strain was 0.1% while the maximum strain applied to the sample was 3%. In the strain sweep measurements, the frequency used was also 3.14 rad/s (0.5 Hz) 6.28 rad/s (1 Hz); for consistency purposes, the strain range examined was from 0.01% up to 100% and the results were documented with a rate of 5 data points per order of magnitude. For the steady-state flow step measurements, the strain range examined was also 0.01% to 100%, and the measurement rate was also 5 data points per order of magnitude while each measurement ran for 0.5 min. Calibration samples (standard oils) were also run, which were found to have values within 5% of their expected values, thus proving the good operation of the instrument.

### 2.12. Docking Procedures

Three docking approaches have been utilized for the docking of the peptides to the receptor dimer.

HADDOCK: Haddock [22] requires the co-ordinates of both the receptor and the ligand. In our case, the receptor is the extracellular domain of the TβRII-TβRI complex, and the ligand is either the hexapeptide (peptide 6) or the octapeptide (peptide 8). Since peptides are very flexible, we derived their structures from the trajectories of 20 ns MDS after clustering and adopting the representative structures from the cluster with the highest population. HADDOCK provided us with 200 models for the complex of TβRII-TβRI with each one of the resulted peptide 6t or peptide 8t conformations. Next, the docking models have been screened for those with the peptide bound in the cleft between TβRII and TβRI (here called centric models). The centric model with the lowest ΔG, as calculated by PRODIGY [23], has been chosen as the most probable one, one for each peptide form. Since backbone flexibility is also allowed to some degree, we tested in the docking results the integrity of disulphide bonds, which are present in the crystal structure of the receptor molecules. Finally, the resulted complexes have been subjected to MDS as described in Materials and Methods.

HPEPDOCK: HPEPDOCK [24] generates its own ensemble of peptide conformations starting with peptide structures, as well as sequences. It also expects the structure of the receptor as input, which is treated as rigid. HPEPDOCK provided us with 100 models. Again, a centric model with the lowest ΔG has been identified as the most probable one for peptide 8t and peptide 6t. The resulted complexes have been subjected to MDS.

MDOCKPEP: MDockPeP server predicts a protein–peptide complex, accepting as input the receptor structure and the peptide sequence [25]. The server allows a degree of backbone flexibility. We used the top ten models for affinity calculations and identified the most stable one (most negative ΔG), which has been subjected to MDS.

### 2.13. Molecular Dynamics Simulations

Models of TβRII-TβRI complexes with bound peptide 8 or peptide 6 derived from the three docking procedures, as well as the TβRII-TβRI dimer itself, have been subjected to 20 ns equilibration MDS at constant volume and temperature (300 K) using NAMD2 [26] with the Charmm36 force field for proteins. Similar simulations have been conducted also for the (TβRII-TβRI)-TGF complex, both free and with bound peptide 8t or peptide 6t. The simulation boxes were prepared with VMD [27] to contain TIP3 water and charge-neutralizing NaCl at a concentration of 0.15 M. Average affinities over the last 200 frames of the ten molecular dynamics trajectories have been calculated using PRODIGY [23] In addition, the flexibility of TβRII and TβRI backbones over the above simulations has been calculated in terms of Cα atom rmsf (root mean square fluctuations) using wordom [28].

### 2.14. Statistical Analysis

Results are presented as mean ± SD of three independent experiments unless otherwise stated. Statistical differences between experimental groups were determined by one-way ANOVA and values of *p* < 0.05 were considered significant. All the analyses were performed by using GraphPad Prism 8. Quantification of Western blots was performed by ImageJ 1.53t.

## 3. Results

### 3.1. Overproduction and Purification of L83-S112 C-Terminal Domain of TGF-β1

**Figure 1:**
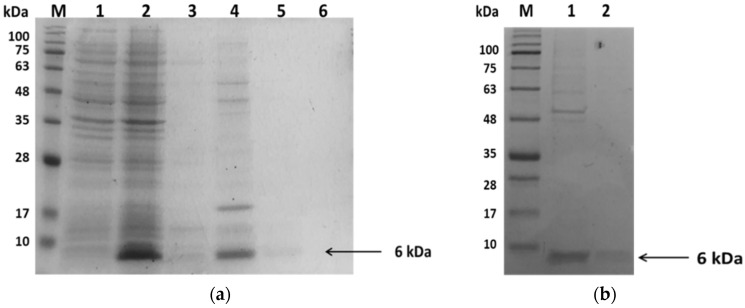
SDS-PAGE analysis in 15% w/v polyacrylamide gel: (**a**) of overexpressed and purified L^83^-S^112^ protein of the C-terminal domain of TGF-β1; Lane M: protein markers, Lane 1: cell lysate before IPTG addition, Lane 2: cell lysate after IPTG addition, Lane 3: 1st wash, Lane 4: 1st elution, Lane 5: 2nd elution, and Lane 6: 3rd elution. L^83^-S^112^ C-terminal region of TGF-β1 containing 6-His tag is depicted with an arrow. (**b**) FPLC purified L^83^-S^112^ protein of the C-terminal domain of TGF-β1; Lane M: protein markers, Lane 1: 7th elution fraction, and Lane 2: 8th elution fraction.

The L^83^-S^112^ C-terminal TGF-β1 was cloned initially at the pAN5 plasmid vector to fuse the sequence for in vivo biotinylation. The recombinant vector pAN5-L^83^-S^112^ TGF-β1 was used as a template to amplify the sequence with the 14-mere sequence for in vivo biotinylation and cloned to plasmid vector pET-29(+). The truncated protein L^83^-S^112^ C-terminal region of TGF-β1 containing the 6-His tag at the carboxy terminus and the sequence for in vivo biotinylation at the amino terminus was overexpressed in *E. coli* strain BL21(DE3). The L^83^-S^112^ C-terminal region of TGF-β1 was purified from inclusion bodies with Ni-NTA column agarose beads. Figure 1 shows the results of overexpression and purification. The desired product is expected at 6 kDa and is detected at sequential elutions (Figure 1a).

To further purify the protein, high-pressure liquid chromatography (Fast Flow Liquid Chromatography, FPLC) (AKTA pure, General Electric, Chicago, IL, USA) with a nickel column was used. The L^83^-S^112^ C-terminal region of TGF-β1 was detected from the 6th elution fraction to the 11th elution fraction. As represented in Figure 1b, the maximum concentration of the protein was observed in the 7th elution fraction, and then, it began to decrease.

### 3.2. In Vitro Chondrogenic Differentiation of hDPSCs Is More Effective When Cells Are Treated with the Peptides Derived from the C-Terminal Domain in Monolayer Cultures

**Figure 2:**
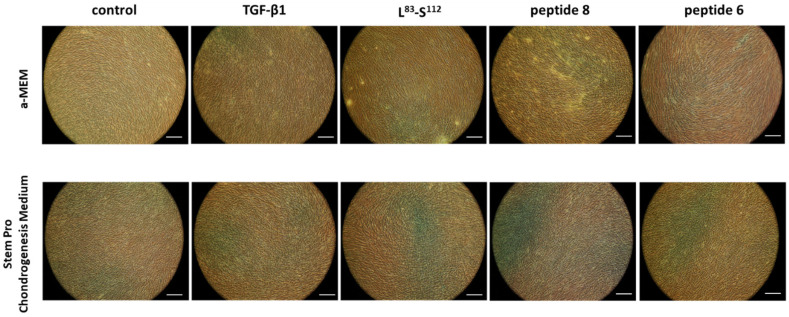
Alcian Blue staining on the 14th day of differentiation of hDPSCs cultured in complete medium alpha MEM or StemPro Chondrogenesis medium with TGF-β1, C-terminal L^83^-S^112^ TGF-β1, peptide 8, or peptide 6. Scale bars: 50 μm.

In order to investigate the chondrogenic capacity of the three peptides, hDPSCs were treated with equimolar concentrations of TGF-β1 or L^83^-S^112^ C-terminal or peptide 8 or peptide 6. The chondrogenic differentiation capacity of L^83^-S^112^ C-terminal domain, as well as peptide 8 and peptide 6, was investigated with Alcian Blue staining after 14 days of differentiation. Data revealed that the three short peptides are functional and can induce in vitro chondrogenesis, with peptide 8 being more efficient that the other two peptides, peptide 6 and L^83^-S^112^ C-terminal domain. As depicted in Figure 2, peptide 8 induced the formation of proteoglycans to a greater extent than the other peptides.

After 14 days of differentiation, Alcian Blue staining was semi-quantified with guanidine hydrochloride by measuring the optical density at 600 nm. The results verified that hDPSCs treated with peptide 8 produce more proteoglycans than hDPSCs treated with other peptides (Figure S1, Supplementary Materials).

### 3.3. In Vitro Chondrogenic Differentiation of hDPSCs Is More Effective When Cells Are Treated with the Peptides Derived from the C-Terminal Domain in 3D Cultures

**Figure 3:**
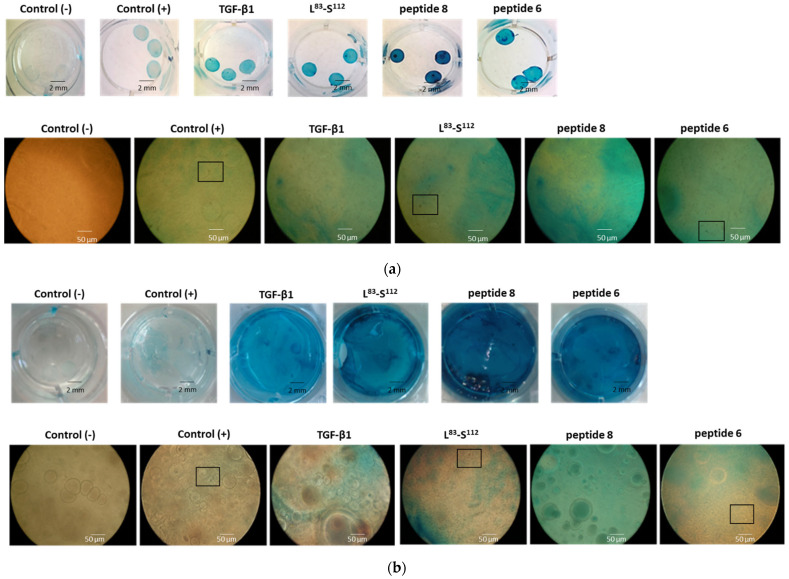
Alcian Blue staining on the 14th day of differentiation of hDPSCs cultured in: (**a**) alginate beads, and (**b**) gelatin type B-alginate gels and treated with TGF-β1, C-terminal L^83^-S^112^ TGF-β1, peptide 8, or peptide 6. Negative control is hDPSCs treated with alpha MEM while positive control is hDPSCs treated with Stem Pro Chondrogenesis Medium. Cells retain their spherical shape and are depicted as black spots within the black boxes. Scale bars are indicated.

The great need to work with models that mimic the function of a living tissue [29] led to the study of the chondrogenic differentiation capacity of hDPSCs treated with full-length TGF-β1 or L^83^-S^112^ C-terminal domain or peptide 8 or peptide 6 in 3D cultures. The encapsulation of hDPSCs in alginate beads and further in vivo differentiation showed that peptides are active upon cells treatment. Peptide 8 exhibited the greatest differentiation capacity, followed by peptide 6 and the L^83^-S^112^ C-terminal region (Figure 3a). Similarly, hDPSCs seeded in gelatin type B–alginate gels and treated with protein and peptides differentiated to chondrocytes, as proteoglycan formation is observed. Peptide 8 exhibited the highest amount of proteoglycan formation (Figure 3b). The results were also verified by measuring the optical density at 600 nm of Alcian Blue staining (Figure S2, Supplementary Materials).

### 3.4. In Vitro Chondrogenic Differentiation of hDPSCs Affects the Rheological Properties of the Gelatin Type B–Alginate Gels

Initially, the rheological properties of hDPSC suspensions in alpha MEM were studied on their own, i.e., without a scaffold, as a function of cell concentration, in a window of cell concentrations relevant to this study and using two types of experimental setups.

Firstly, the dynamic viscosity of each cell suspension was measured as a function of shear rate (Figure S3a). The highest cell concentration suspension (10^8^ cells/mL) exhibits a η90 of ca 1.8 mPas, i.e., only twice the value of η90 of the cell-free, pure alpha MEM solvent. As the cell concentration in the suspension decreases, its viscosity decreases as well, approaching the viscosity of the pure solvent. The dynamic viscosity is also seen to decrease with increasing shear rate, displaying shear-thinning behavior, a characteristic of non-Newtonian fluids. This behavior arises from the intercellular interactions, as well as the cellular deformations, taking place in the shear field. Cell aggregates that may initially exist in the suspension become gradually destabilized and aligned upon application of an increasing shear field, leading to the gradual decrease of the viscosity and the observed shear-thinning effect. Secondly, oscillatory measurements were carried out as a function of time (Figure S3, Supplementary Materials). Only noise was obtained at all concentrations, showing the absence of measurable viscoelastic properties of the cell suspensions at all cell concentrations.

**Figure 4:**
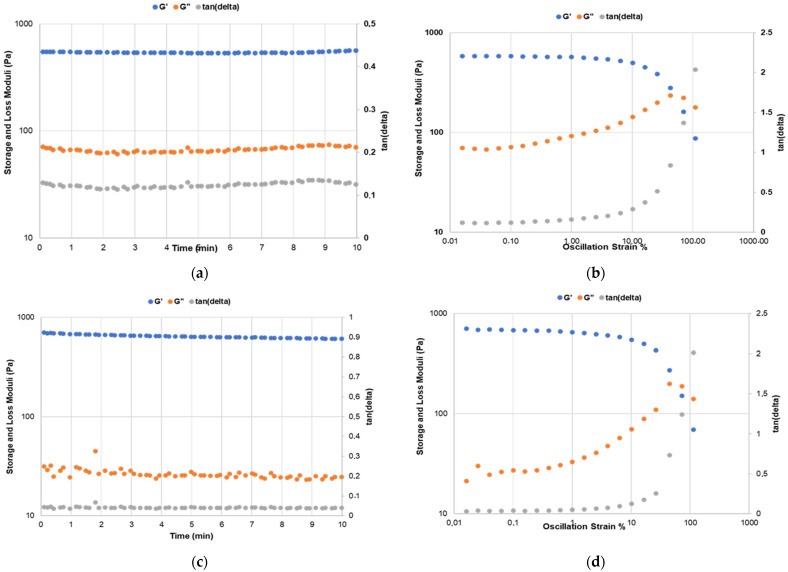
Oscillatory rheological measurements of cell-free and cell-laden hydrogels at different incubation times in cell culture conditions: representative time sweep measurements of cell-free hydrogel at time 0 (**a**) and of cell-laden hydrogels following 14 days of incubation (**b**); representative strain sweeps of cell-free hydrogel at time 0 (**c**) and of cell-laden hydrogels following 14 days of incubation (**d**).

Subsequently, self-supporting, stable gelatin type B–alginate hydrogels were prepared in alpha MEM and their rheological properties were measured at time 0 (fresh) and after 14 days in the cell culture (Figure 4 and Table 4). Crucially, both oscillatory strain sweep and time sweep measurements within LVR yielded thoroughly consistent and reproducible data in all cases and at all time points. The hydrogels are found to display classic viscoelastic behavior with G′ > G″ at the measured frequency, demonstrating the establishment of a continuous, strong, physical, semi-rigid, three-dimensional fibrous network in alpha MEM. The measured elastic modulus of the hydrogel is due almost exclusively to its alginate component, since it is known that the gelatin chains do not have the ability to form a stable three-dimensional network at the conditions employed during these cell culture experiments.

In the absence of cells, the hydrogels are seen to change during their existence in actual cell culture conditions for 14 days (Table 4). In particular, at the end of the 14 days, the hydrogels are characterized by an increase of the elastic modulus of ca 10% with a concomitant drop of the viscous modulus G″ by ca 55%, compared to the average values of G′ and G″, respectively, at time 0. This shows that the cell-free hydrogels slowly remodel during their incubation in actual cell culture conditions, ending up with a more mature and stronger three-dimensional alginate network structure, which has, however, lost its non-bound biopolymer chains (mainly collagen), possibly via diffusion and/or hydrolysis, thus leading to the observed significantly decreased viscous moduli at the end of the 14 days (Table 4).

Similar comparative oscillatory rheological studies of the hydrogels were conducted following the three-dimensional encapsulation of dental pulp stem cells within the hydrogels (Table 4 and Figure 4). At time 0, the cell-laden hydrogels are found to possess the same qualitative and quantitative rheological characteristics as their cell-free counterparts. Therefore, at time 0, the presence of cells within the hydrogels does not seem to affect the values of G′ and G″; this is in complete agreement with our findings with the cell suspensions (Figure S3), thus demonstrating once more that the mere presence of the cells themselves do not play a measurable role on the viscoelastic parameters of the sample.

Following incubation in identical, actual cell culture conditions for 14 days of both cell-free and cell-laden hydrogels, in both cases, in the absence of the growth factor peptide, it is found that the cell-laden hydrogels display on average an increase of their elastic character by ca 20% compared to their cell-free counterparts; furthermore, it is observed that the cell-laden hydrogels display, on average, an increase of their viscous character by ca 180% (i.e., it almost triples!) compared to their cell-free counterparts (Table 4 and Figure 4). We propose that this significant improvement of the rheological properties of the hydrogels in the presence of encapsulated cells is due to the metabolic activity of the cells, specifically the production of new ECM by the cells during the 14-day incubation period; in addition, based on the evidence presented here, it seems that the newly produced ECM ingredients have a predominant viscous rather than elastic character under the specific experimental conditions.

In parallel, cell-laden hydrogels were incubated in the presence of the peptide growth factor and compared with the cell laden hydrogels incubated in the absence of peptide growth factor, in otherwise identical cell culture conditions (Table 4 and Figure 4). It is observed that the cell-laden hydrogels incubated in the presence of the peptide growth factor have ca 10% lower elastic modulus G′ and ca 90% lower viscous modulus G″, compared to the cell-laden hydrogels incubated in the absence of the peptide growth factor. These data provide evidence that the presence of peptide 8 in the cell culture medium influences in a measurable manner the rate of the cellular metabolic activity and/or the transduced metabolic pathways, thus leading to the production of different amounts and/or different types of ECM components during the incubation period.

### 3.5. Implication of TGF-β1 Functional Peptides in Extracellular Matrix Homeostasis in Monolayer Cultures

**Figure 5:**
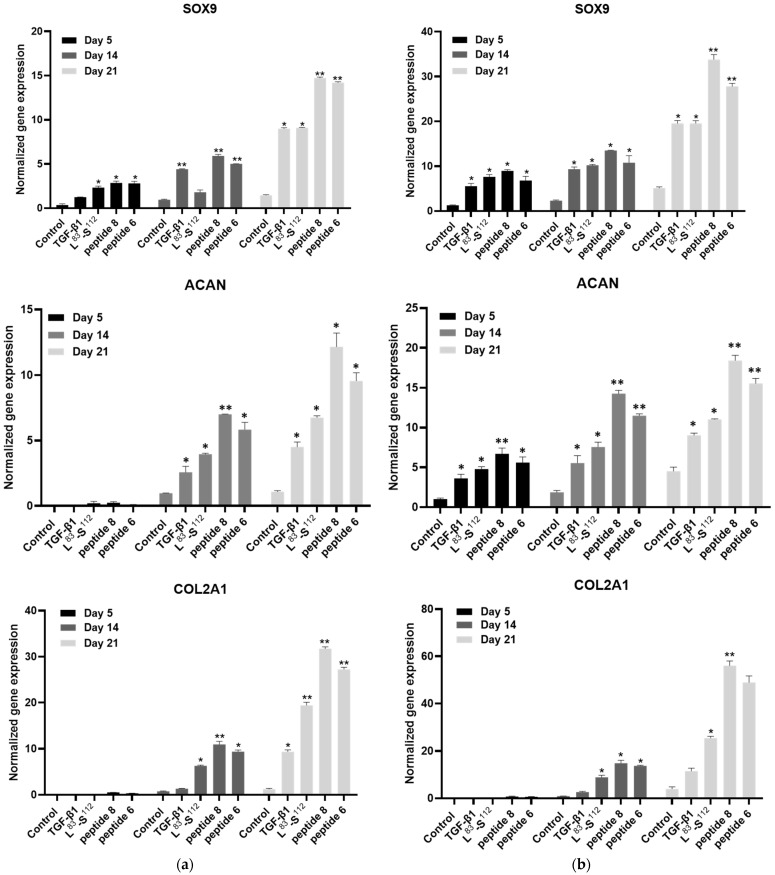
Relative expression levels of *SOX9, ACAN,* and *COL2A1* on 5th, 14th, and 21st day of chondrogenesis in monolayer culture (**a**) alpha MEM and (**b**) Stem Pro Chondrogenesis medium. *GUSB* and *GAPDH* were used as reference genes. Error bars represent the ± SD of three independent experiments. Asterisks (*) and (**) indicate the statistical significance (*p* ≤ 0.05 and *p* ≤ 0.01, respectively) compared to control cells.

The balance between ECM formation and degradation was investigated by quantifying the expression levels of *COL2A1, ACAN COL1A1, COLX, ALP, RUNX2, SOX9, ADAMTS-5,* and *MMP13.* Total RNA was isolated at the 5th, 14th, and 21st day of differentiation from hDPSCs treated with full-length TGF-β1 or L^83^-S^112^ C-terminal domain or peptide 8 or peptide 6. qPCR analysis showed that collagen type II expression increased over differentiation time and, along with the aggrecan expression and proteoglycan formation, indicate an induction of ECM formation. Specifically, treatment with peptide 8 resulted in the upregulation of the chondrogenic markers *COL2A1, ACAN,* and *SOX9* in both culture media. In addition, cells cultured in the Stem Pro Chondrogenesis medium presented a higher expression of the chondrogenic markers compared to alpha MEM. Peptide 6 and L^83^-S^112^ C-terminal domain increased the expression of the chondrogenic markers; however, the increase was lower compared to peptide 8 (Figure 5).

**Figure 6:**
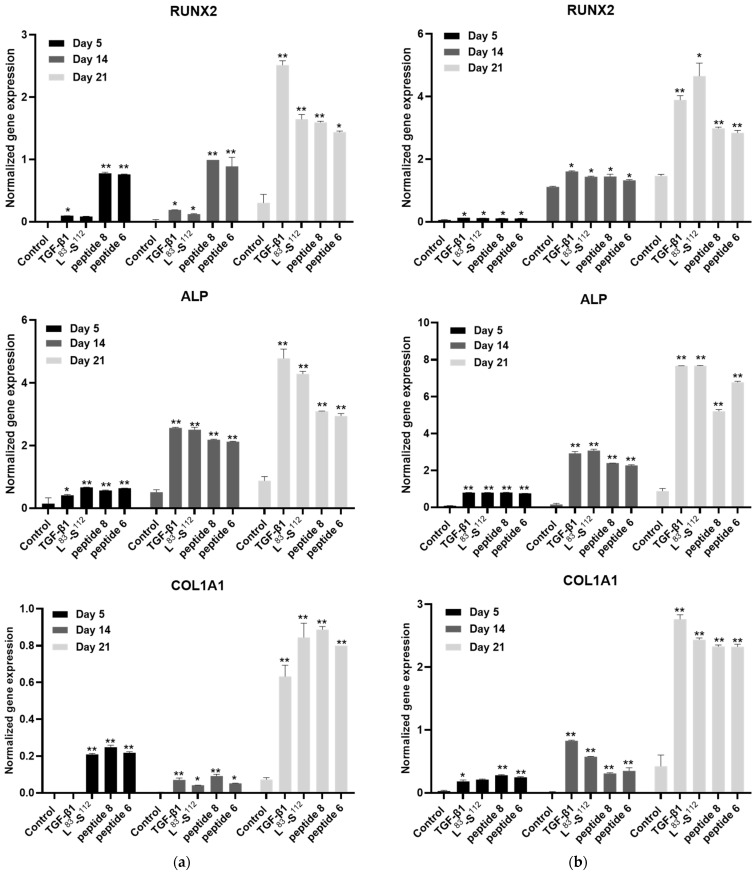
Relative expression levels of *RUNX2, ALP, COL1A1,* and *COL2A1* on 5th, 14th, and 21st day of chondrogenesis in monolayer culture (**a**) alpha MEM and (**b**) Stem Pro Chondrogenesis medium. *GUSB* and *GAPDH* were used as reference genes. Error bars represent the ± SD of three independent experiments. Asterisks (*) and (**) indicate the statistical significance (*p* ≤ 0.05, and *p* ≤ 0.01, respectively) compared to control cells.

In order to investigate the possibility of chondrocyte dedifferentiation and osteogenic differentiation, the expression levels of both were quantified. Treatment of cells with the peptides showed a decrease in the gene expression in alpha MEM media, while, in Stem Pro Chondrogenesis medium, among the peptides, only peptide 8 showed a higher decrease in *COL1A1, ALP,* and *RUNX2* compared to the full-length protein (Figure 6).

**Figure 7:**
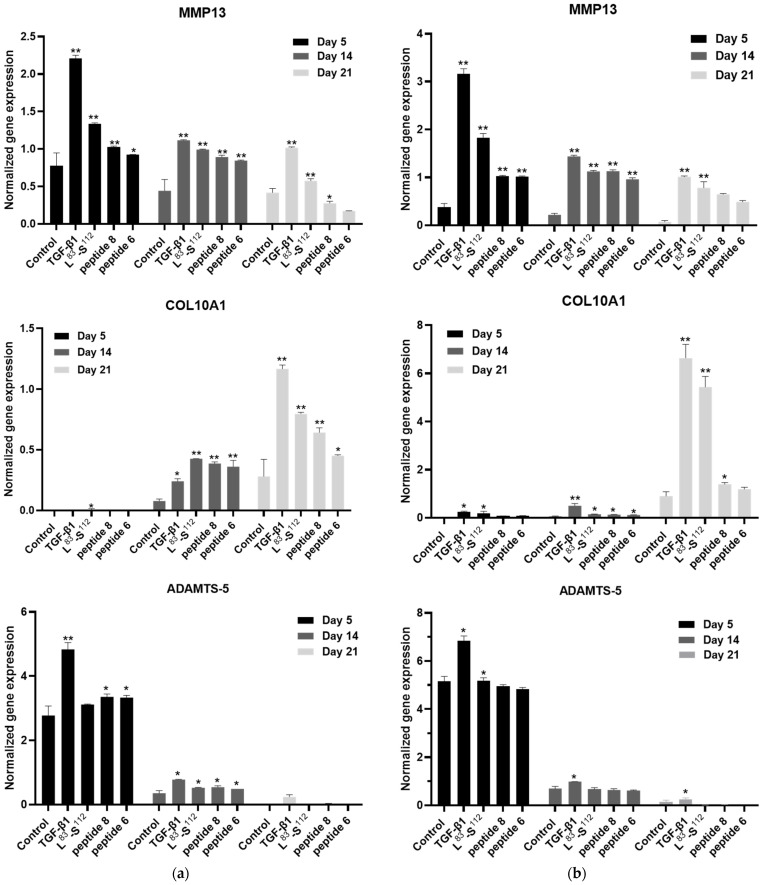
Relative expression levels of *MMP13, ADAMTS-5,* and *COL10A1* on 5th, 14th, and 21st day of chondrogenesis in monolayer culture (**a**) alpha MEM and (**b**) Stem Pro Chondrogenesis medium. *GUSB* and *GAPDH* were used as reference genes. Error bars represent the ± SD of three independent experiments. Asterisks (*) and (**) indicate the statistical significance (*p* ≤ 0.05, and *p* ≤ 0.01, respectively) compared to control cells.

As aforementioned, exogenous cell treatment with TGF-β1 alters metalloproteinase expression, and, if upregulated, it leads to ECM degradation. ECM turnover is also verified by the upregulation of *COL10A1*, a hypertrophic marker. To further investigate the effect of exogenous cell treatment with the short peptides, *ADAMTS-5, MMP13,* and *COL1OA1* expression were studied. *ADAMTS-5* shows its maximum expression on the 5th day of differentiation and its levels gradually decrease over time. Treatment with peptides lead to a decreased expression. Moreover, the same decline is observed for *MMP13*. hDPSCs treatment with peptide 6 exhibited the greatest decrease in *ADAMTS-5* and *MMP13* expression levels, followed by peptide 8, L^83^–S^112^ C-terminal TGF-β1, and full-length TGF-β1. The same pattern of gene expression is observed for cells cultured in Stem Pro Chondrogenesis medium. (Figure 7a,b).

**Figure 8:**
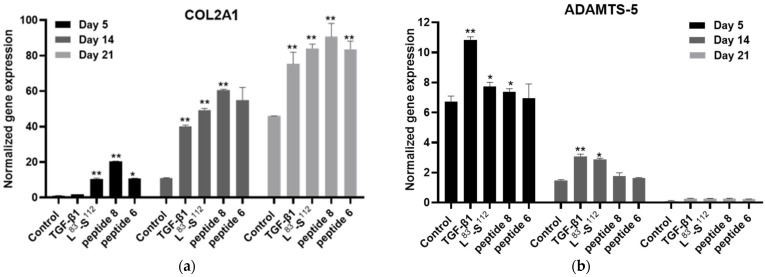
Relative expression levels of (**a**) *COL10A1* and (**b**) *ADAMTS-5* (on 5th, 14th, and 21st day of chondrogenesis in 3D culture. *GUSB* and *GAPDH* were used as reference genes. Error bars represent the ± SD of three independent experiments. Asterisks (*) and (**) indicate the statistical significance (*p* ≤ 0.05, and *p* ≤ 0.01, respectively) compared to control cells.

In 3D cultures, i.e., alginate beads, *COL2A1* expression increased after treatment with the peptides, indicating that the 3D structure affects cell proliferation, differentiation, and, subsequently, ECM formation. Concerning *ADAMTS-5* expression, upon treatment with the peptides, a lower expression was observed compared to the full-length protein. Among the different peptides, peptide 6 exhibited the highest reduction (Figure 8).

### 3.6. Interaction of the Peptides with the Receptors

#### Hypothesis Testing

We test, computationally, the following hypothesis, as suggested by our experiments:

The symmetrical extracellular TGF binding to the two receptor dimers TβRII-TβRI can induce reactions of their intracellular domains more effectively, if the receptor dimers bound peptide 8 (and, less effectively, peptide 6).

According to the crystal structure (PDB: 3KFD) of the (TβRII-TβRI)-2TGF-(TβRII-TβRI) extracellular domain complex, the biological unit is a hexamer. Keeping in mind that TβRII and TβRI are transmembrane proteins with big interacting intracellular domains, the TGF dimer seems to both stabilize the TβRII-TβRI dimer and hold the two receptor dimers at a specific distance. It seems very likely that the concrete extracellular arrangement of TGF with its partner molecules favors specific modes of interaction of the intracellular TβRII and TβRI domains. This scenario can be realized in two versions, (a) and (b), as follows:

(a) TGF is absent. In this case, the peptide can bind to the TβRII-TβRI complex, stabilizing it. This can be carried out if the peptide can bind to both subunits at the same time, e.g., in the space between the two subunits. Therefore, our docking results should find such associations more stable than TβRII-TβRI alone, which turned out to be the case. This mode of peptide action promotes the interaction of the intracellular domains of TβRII and TβRI and the subsequent activation of the type I receptor kinase. Since reliable structural models of the full-length receptors (extracellular + transmembrane + intracellular) are not yet available, it is not possible to argue about further interactions between the intracellular domains of the dimers. Nevertheless, since more TβRII-TβRI dimers will be present with a peptide bound than without, a higher outcome of the Smad pathway branch is expected. TβRII-TβRI dimers can also approach each other, and they may interact intracellularly in several modes. One of these modes could be similar to that dictated by TGF. Moreover, peptide binding has a kinetic advantage over TGF. Ignoring the lateral diffusion of the receptors, one can estimate the ratios of the translational (Dt,pept/Dt,TGF) and rotational (Dr,pept/Dr,TGF) diffusion constants of the peptides and TGF using the HullRad software version 9 [30]. This calculation shows that the peptides reach their target 4 times faster and may scan the binding sites rotationally 60 times faster than the TGF dimer does.
(b) TGF is present. In this case, the peptide can bind to the (TβRII-TβRI)-TGF2-(TβRII-TβRI) hexamer at the same receptor intersubunit position as in case (a), enhancing its stability. Our docking results, combined with MDS, should find the hexamer + peptide more stable than the hexamer alone. In order to save simulation time, we actually tested the stability of the (TβRII-TβRI)-pept-TGF1 complex.

Both modes of action (a and b) should differ in their effectiveness using either peptide 8t or peptide 6t, if the peptides bind to TβRII-TβRI with different affinities, which actually is, again, the case. The extracellular binding of the TGF homodimer to the TβRII and TβRI receptors serves the stabilization of the TβRII-TβRI complex and dictates the specific mode of interaction of the intracellular domains between each other, as well as with Smad proteins. In addition, similarly to case (a), differences in the extent of backbone fluctuations of the carboxyl termini, nearest to the transmembrane helix, could provide a hint about differences in the transmitted signals to the intracellular domains.

Since peptides are very flexible, we expect many of their different conformations to be present in the vicinity of the receptor complex (TβRII-TβRI) before binding. Therefore, more than one peptide conformation has been tested as docking candidates. Among the docking results, we considered for further computation only those with the peptide intercalating between TβRII and TβRI. An analysis of our docking and MDS results suggests that the peptides can bind in the cleft between the subunits of the TβRII-TβRI dimer, conferring its stability, as it becomes obvious when comparing the affinities for the formation of TβRII-TβRI with any of its peptide-docking models from HADDOCK, HPEPDOCK, and MDOCKPEP (Table 5). Moreover, the complexes with peptide 8 are consistently more stable than those with peptide 6. As expected, binding with the TGF dimer has a much higher stabilizing effect than with the peptides alone and it is strengthened by the presence of the peptides. It is worth noting that, during the MDS, the peptide constantly changes its conformation, as well as its position, contacting, however, both TβRII and TβRI. The amino acid composition of the peptides’ binding pockets (4.5 Å from peptide atoms) at the end of the 20 ns simulations are given in Table 6. In average, over all docking programs (including the complexes with TGF), peptide 6 makes ~44% more contact with TβRI than with TβRII. On the other hand, peptide 8 shows no clear preference.

**Figure 9:**
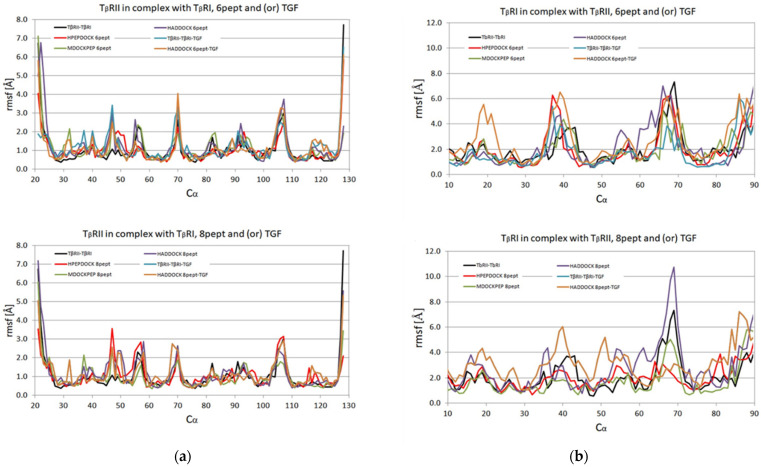
Backbone fluctuations of (**a**) TβRII derived from MDS of the TβRII-TβRI complex, and (**b**) TβRI derived from MDS of the TβRII-TβRI complex. The black profile refers to the peptide-free complex. Bound states with peptide 6 (**a**) and peptide 8 (**b**), as well with TGF, resulting from docking models of HPEPDOCK, MDOCKPEP, and HADDOCK, are denoted in color (6pep: peptide 6 and 8pep: peptide 8).

Beside conformational changes, backbone fluctuations can also transmit signals through the transmembrane helix. Therefore, we also tested the effect of peptide binding on the dynamics of the receptors’ backbone, especially the differences in the extent of the Cα atom fluctuations (rmsf) of the carboxyl termini. More intense backbone fluctuations are observed in the case of peptide 6 binding and, especially, upon binding to TβRI. The results are visualized in Figure 9a,b.

**Figure 10:**
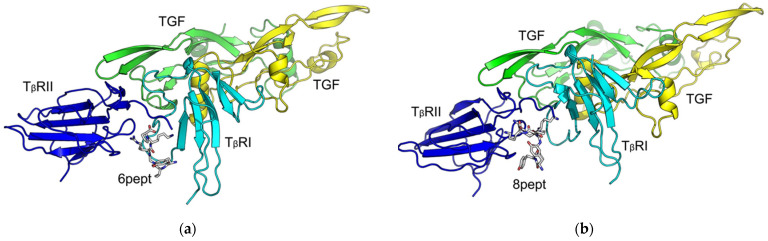
Representation of the HADDOCK docking models for the complex TβRII-TβRI-peptide-TGF2 after MDS: (**a**) for peptide 6 and (**b**) for peptide 8. Blue: TβRII; Cyan: TβRI; Green and Yellow: TGF; and Grey: Peptide. The image has been produced with PyMol [31].

As previously mentioned, the peptides can bind in the cleft between the subunits of the TβRII-TβRI dimer, conferring its stability. Figure 10 represents the HADDOCK docking models for the complex TβRII-TβRI-peptide-TGF2 after MDS for peptide 6 (Figure 10a) and for peptide 8 (Figure 10b).

### 3.7. Peptides Promote the Activation of the MAPK and Smad2 Pathways

**Figure 11:**
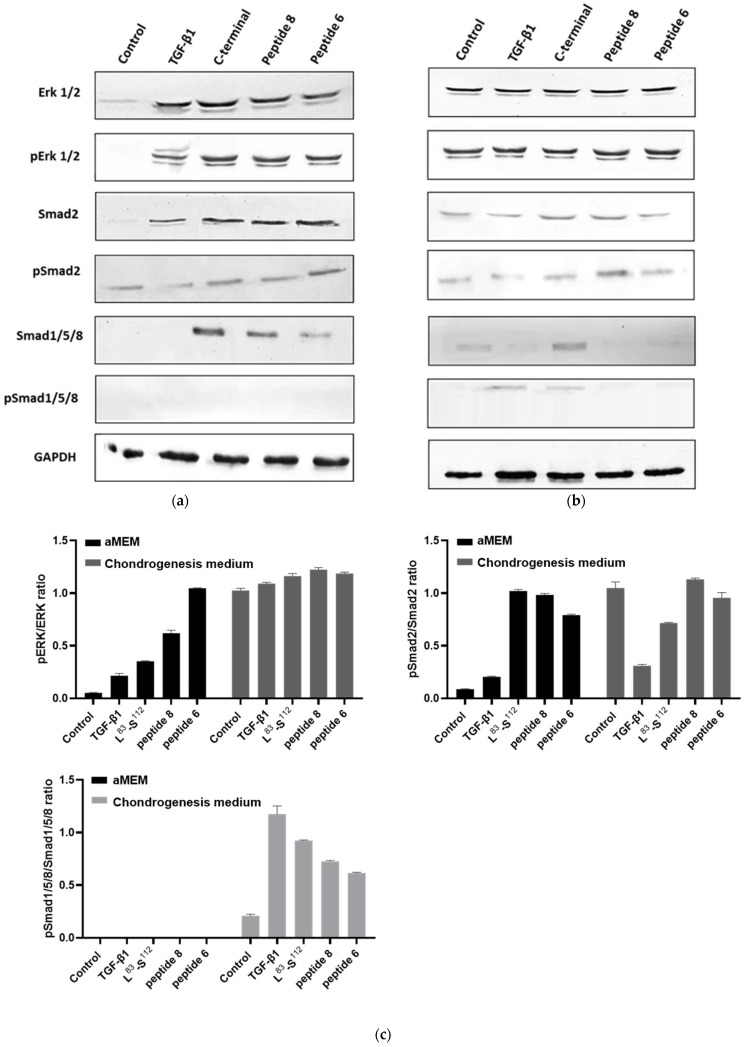
Representative immunoblots of ERK1/2, pERK1/2, Smad2, pSmad2, Smad1/5/8, and pSmad1/5/8 (**a**) in alpha MEM and (**b**) StemPro Chondrogenesis medium in monolayer culture at the 21th day of differentiation. (**c**) Quantitative analysis of ERK1/2, Smad2, and Smad1/5/8 phosphorylation in hDPSCs after in vitro chondrogenesis induction with TGF-β1 and its peptides. Error bars represent the ± SD.

The involvement of the MAPK pathway and Smad2 pathway in modulating the chondrogenic differentiation of the stem cells has been demonstrated previously [32]. TGF-β1 induces chondrogenesis through the canonical Smad2 pathway, resulting in the upregulation of collagen type 2. The effects of the peptides on the canonical and non-canonical pathway were studied by immunoblotting with ERK1/2, pERK1/2, Smad2, pSmad2, Smad1/5/8, and pSmad1/5/8 antibodies at the 21th day of differentiation. In Figure 11a, peptides activated the canonical pathway because they increased the phosphorylation level of ERK and Smad2. In the Stem Pro Chondrogenesis medium, treatment with peptides also increased the phosphorylation level of ERK and Smad2, and peptide 8 mainly increased the phosphorylation of Smad2. In this case, TGF-β1 increased the levels of phosphorylated Smad1/5/8 as indicated by the ratio.

## 4. Discussion

The maintenance of articular cartilage depends on the balance between anabolic and catabolic processes, mediated by different cytokines. Among them, TGF-β-mediated anabolic signaling represents a critical aspect of the articular cartilage life cycle and homeostasis. More specifically, the three TGF-β isomorphs play a crucial role in chondrogenesis, a well-orchestrated process that involves mesenchymal condensation, chondrogenic proliferation of chondroprogenitors, and chondrocyte differentiation. The isoform ΤGF-β1 regulates the proliferation and differentiation of chondroprogenitors to chondroblasts [33].

The active dimer TGF-β1 binds to the heteromeric receptor complex and triggers cell signaling that regulate the extracellular matrix structure. The interaction of TGF-β1 with ALK1 promotes a catabolic response by causing the phosphorylation of Smad-1/5/8, followed by a positive regulation of the transcription of metalloproteinase genes such as *MMP13* and *ADAMTS-5*. Upon interaction with the ALK5 receptor, it induces the phosphorylation of Smad2 and -3, promoting proteoglycan synthesis and an anabolic response [34]. The interaction with the receptor takes place through the C-terminal region and the critical amino acids for this process are Arg25, Lys31, Trp32, His34, Tyr91, Val92, Gly93, Arg94, and Val98 [35,36,37]. From molecular simulation experiments (unpublished data), three short peptides of the C-terminal domain were designed and investigated against their functionality. L^83^-S^112^ C-terminal ΤGF-β1 was overproduced and purified from inclusion bodies while the two short peptides were chemically synthesized.

hDPSCs treated with different proteins and peptides, as concluded from Alcian Blue staining and the relative expression of chondrogenic markers, *COL2* and *SOX9*, differentiated into chondrocytes because of proteoglycan and collagen type II formation. Therefore, peptides remained active andresulted in the induction of in vitro chondrogenesis in both monolayer and 3D cultures. As indicated by the qPCR analysis among the three peptides, results showed that peptide 8 induces chondrogenesis to a greater extent than peptide 6 and the L^83^-S^112^ C-terminal region, resulting in an upregulation of *COL2A1* and *SOX9*. The increase in *COL1A1, RUNX2,* and *ALP* expression after treatment with the full-length protein has also been reported by Bai et al. [38]. In our study, peptides compared to the full-length protein decreases the expression of these markers indicating an induction of chondrogenic differentiation. Moreover, peptide 8 and peptide 6 decreased the expression of *COL1A1, MMP13,* and *ADAMTS-5* proteins involved in ECM degradation and hypertrophy.

The capacity of peptide 8 to promote chondrogenic differentiation has been reported after incorporation to fibrous scaffolds [39]. Similar to the monolayer culture, peptide 8 demonstrated the greatest differentiation capacity in alginate beads. Proteoglycan synthesis and collagen type II expression were increased in 3D cultures of hDPSCs, a phenomenon that is supported for other cells lines, like bone-marrow-derived hMSCs [40]. Moreover, ECM synthesis is supported by the differences observed in the rheological properties of the gelatin type B–alginate gels. In this case, differentiated cells (treated with peptide 8) affected the rheological properties of the gels in a measurable manner, indicating the production of different amounts and/or different types of ECM components during the incubation period.

The greater differentiation capacity of the short peptides is probably because they adopt the appropriate configuration for binding to the ALK5 receptor. According to molecular dynamic simulations, TβRI is more flexible than TβRII and both peptides can affect the dynamics of the receptors’ backbones. Specifically, the flexibility of residues 45–47 of TβRI, which do not belong to the binding site, is enhanced by both peptide 6 and peptide 8 binding. On the contrary, the effect is reversed concerning the carboxyl ends. The effect is more intense with TβRI. Residues 16–22, 35–42, and 49–58 belong to loop regions distant from the binding site and their mobility is strongly affected by peptide binding. In this case, the flexibility of the carboxyl end is also rather enhanced than suppressed. One can speculate that changes in the dynamics of TβRII-TβRI upon peptide binding may be detected by the intracellular domains, leading to differences in function. The effect of the peptide binding on the backbone dynamics of the receptors is also of interest, since dynamic signals can be transmitted through the transmembrane helical domain to the intracellular domains affecting their state. As mentioned above, a reliable model of the full hexamer is not yet feasible. However, it is possible to apply molecular dynamics simulations (MDSs) on the extracellular domains alone and compare their backbone dynamics in the presence and in the absence of the peptides. Especially, differences in the extent of backbone fluctuations of the carboxyl termini, nearest to the transmembrane helix, could provide a hint about differences in the transmitted signals to the intracellular domains. The C-terminal region (L^83^-S^112^), although possessing the necessary amino acids for binding to the receptor, may not adopt the most favorable configuration for rapid binding to the receptor.

**Scheme 1:**
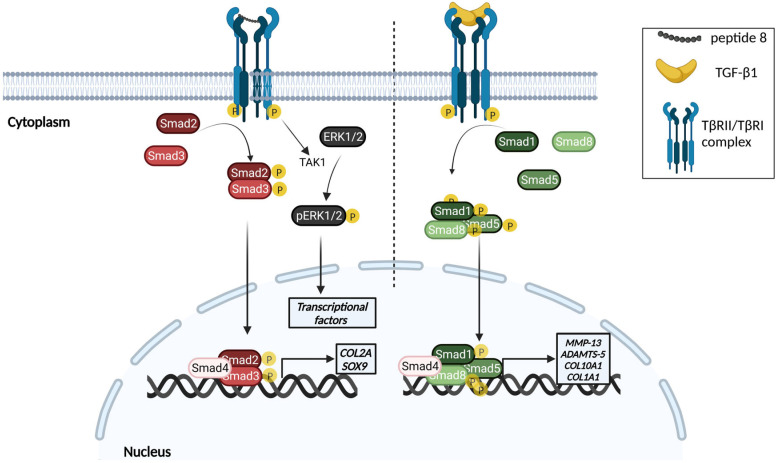
Schematic representation of the main research findings. MDS data propose that peptide 8 favors and stabilized binding of the endogenous full-length protein to the receptor complex and induces the ERK1/2 and Smad2/4 signalling cascades, which regulate the expression of genes related to the extracellular matrix regeneration.

In correlation with the qPCR and MDS data, binding to ALK5 receptor and induction of the anabolic cascade are also supported by the Western blot results. TGF-β1 activates the p38/MAPK pathway, as previously reported by Ma et al., while peptides 8 and 6 promote both the ERK and Smad2/4 pathways, as indicated by the phosphorylated/non-phosphorylated protein ratio. Data show that TGF-β1 also promotes the Smad1/5/8 pathway, explaining the upregulation of *ADAMTS-5* and *MMP13* and the higher expression of *COL1A1, COL10A1, ALP*, and *RUNX2* [41,42,43]. Nevertheless, the short peptides induced a lower expression of *ADAMTS-5* and *MMP13* by ~30% compared to the full-length growth factor. These results, together with proteoglycan and collagen type II synthesis, may propose the idea that short peptides promote extracellular matrix synthesis and decrease extracellular matrix degradation, and, consequently, may be a therapeutic approach for osteoarthritis. A schematic representation of the findings is depicted below (Scheme 1).

## 5. Conclusions

In conclusion, short peptides of the C-terminal region of TGF-β1 remained functional and induced in vitro chondrogenesis of hDPSCs either in monolayer or in 3D cultures. Peptide 8, YYVGRKPK, of the C-terminal region of TGF-β1 was found to have the highest differentiation capacity by increasing *COL2A1* expression and proteoglycan formation. At the same time, the decreased expression of *ADAMTS-5* and *MMP13* indicates that short peptides act as anabolic regulators and promote new ECM formation and do not transition to the hypertrophic phenotype. The proposed mechanism of the peptide’s action relies on binding to the receptors and activating the ERK1/2 and Smad2/4 signalling cascades. which induces the expression of genes related to cartilage regeneration (Scheme 1). Chondrogenesis induction and inhibition of extracellular matrix degradation were found to be better when hDPSCs were cultured in 3D scaffolds and, thus, we propose that the culture method affects the differentiation process. Our findings provide some basis for the development of osteoarthritis treatment using hDPSCs and short functional peptides in the future.

## Figures and Tables

**Table 1:** Primer pairs, digestion enzymes, and plasmid vectors used for amplification and cloning.

Primer Sequence (5′ to 3′)	Restriction Enzyme	Plasmid Vector
5′-CCGCCATATGTCCGGC CTGAACGACATCTTCG-3΄ Forward	NdeI	pET-29c(+)
5΄-CCGCAAGCTTCTAGTG GATGTGGTGTCAGCTCACTTGCAGGAGCG -3΄Reverse	HindIII	pET-29c(+)

**Table 2:** Amino acid sequence of the full-length protein, C-terminal region, and synthetic peptides of TGF-β1.

	Amino Acid Sequence
Full-length mature TGF-β1	ALDTNYCFSSTEKNCCVRQLYIDFRKDLGWKWIHEPKGYHANFCLGPCPYIWSLDTQYSKVLALYNQHNPGASAAPCCVPQALEPLPIVYYVGRKPKVEQLSNMIVRSCKCS
C-terminal region L^83^-S^112^	LEPLPIVYYVGRKPKVEQLSNMIVRSCKCS
Peptide 8	YYVGRKPK
Peptide 6	YVGRKP

**Table 3:** Primer pairs used for qPCR analysis of *GUSB*, *GAPDH*, *COL2A1*, *ADAMTS-5*, *MMP13*, *COL1A1*, *COL10A1*, *RUNX2*, *SOX9*, *ALP*, and *ACAN* gene expression.

Gene	Forward (5′ to 3′)	Reverse (5′ to 3′)
*GUSB*	CATTTTGTCGGCTGGGTGTG	GCCATGGCTTGACGTTGAA
*GAPDH*	GCACCGTCAAGGCTGAGAAC	TGGTGAAGACGCCAGTGGA
*COL2A1*	CAACCAGGACCAAAGGGACA	ACCTTTGTCACCACGATCCC
*ADAMTS-5*	TGGAAGTGAGCAAGAACGCT	ATGCCCACATAAATCCTCCCG
*MMP13*	AAGGAGCATGGCGACTTCT	TGGCCCAGGAGGAAAAGC
*COL1A1*	CAGTGTGGCCCAGAAGAACT	CCGCCATACTCGAACTGGAAT
*COL10A1*	AGAATCCATCTGAGAATATGC	CCTCTTACTGCTATACCTTTAC
*RUNX2*	TCATGGCGGGTAACGATGAA	GTGAAACTCTTGCCTCGTCC
*SOX9*	GGCAAGCTCTGGAGACTTCTG	CCCGTTCTTCACCGACTTCC
*ALP*	CCGAGATACAAGCACTCCCA	TCCGTCACGTTGTTCCTGTT
*ACAN*	GGAAGGGAGGGGAACCATTG	TGATGGCTGTCCACTGACAC

**Table 4:** Summary of the average values of G′ and G″ parameters of hydrogels with and without cells and growth factor peptides at the start and at the end of the cell culture experiments at 37 °C.

**Day 0**		
	**Without cells**	**Untreated cells**
G′ (Pa)	1597 ± 25	1556 ± 28
G″ (Pa)	262 ± 35	278 ± 38
**Day 14**		
	**Without cells**	**Untreated cells**
G′ (Pa)	1750 ± 35	1926 ± 39
G″ (Pa)	116 ± 25	227 ± 17

**Table 5:** Affinities of TβRII-TβRI complexed with peptide 6, peptide 8, and/or TGF2.

u	Complex	Model	<ΔG> [kcal/mol] *
HADDOCK	TβRII-TβRI-peptide 6	163	−14.1
	TβRII-TβRI-peptide 8	140	−15.1
HPEPDOCK	TβRII-TβRI-peptide 6	15	−15.5
	TβRII-TβRI-peptide 8	1	−18.5
MDOCKPEP	TβRII-TβRI-peptide 6	9	−16.7
	TβRII-TβRI-peptide 8	10	−17.4
	TβRII-TβRI		−7.3
HADDOCK	TβRII-TβRI-TGF2		−31.3
	TβRII-peptide 6t-TβRI-TGF2		−36.4

* For the calculation of the total affinity of the reaction TβRII + TβRI + pept + TGF2 → TβRII-TβRI-pept-TGF2 using PRODIGY, we assumed the following order of intermediate events: TβRII + TβRI → TβRII-TβRI, TβRII-TβRI + pept → TβRII-TβRI-pept, TGF + TGF → TGF2, TβRII-TβRI-pept + TGF2 → TβRII-TβRI-pept-TGF2. The affinities (<ΔG>, <Kd>) represent averages over the last 200 frames of the 20 ns simulations.

**Table 6:** Amino acid composition of the peptides’ binding pockets.

	Complex	Amino Acid Composition
HADDOCK	TβRII-TβRI-peptide 6	T_β_RII: A21, V22, F24, L27, S117, D118, E119 T_β_RI: C62, A63, P64, S65, S66, K67
	TβRII-TβRI-peptide 6-TGF2	T_β_RII: K23, S117, D118, E119, C120 T_β_RI: E36, C62, A63, P64, K67, T68, G69, S70, V71, T72, T73, T74, S93, S94, P95, G96
	TβRII-TβRI-peptide 8	T_β_RII: K23, F24, P25, Q26, A94, S95, P96, S116, D118 T_β_RI: E36, R58, P64, S65, V71, T72, T73, T74, Y75, P95, G96
	TβRII-TβRI-peptide 8-TGF2	T_β_RII: V22, K23, F24, Q26, P57, Q58, E59, V60, A93, A94, P96, S116, S117, D118 T_β_RI: C62, A63, S66, T74
HPEPDOCK	TβRII-TβRI-peptide 6	T_β_RII: K23, F24, L27, D118, E119 T_β_RI: E36, R58, C62, A63, S65, S66, K67, G69, S70, V71, T73, T74, Y75
	TβRII-TβRI-peptide 8	T_β_RII: V22, K23, F24, P25, Q26, L27, P57, Q58, E59, P96, K97, S116, S117, D118, E119 T_β_RI: R58, A63, P64, S65, S66, K67, G69, S70, V71, T72, T74, Y75, C76
MDOCKPEP	TβRII-TβRI-peptide 6	T_β_RII: A21, V22, K23, F24, Q26, P57, A94, S95, P96, S116, S117 T_β_RI: T73, T74, Y75, Q79, N83, I85, P88, T89, T90
	T_β_RII-T_β_RI-peptide 8	T_β_RII: A21, K23, F24, P25, Q26, P57, P96, S116, S117, D118 T_β_RI: E36, R58, C62, A63, P64, S65, V71, T72, T73, T74, Y75, E86, T89, V91, K92, S93, S94

## Data Availability

The datasets used and/or analyzed during the current study are available from the corresponding author upon reasonable request.

## Supplementary Materials

The following supporting information can be downloaded at: https://www.mdpi.com/article/10.3390/biomedicines11123182/s1, Figure S1: Semi-quantification of Alcian Blue staining on the 14th day of differentiation by measuring the absorbance at 600 nm. Peptide 8 exhibited the highest differentiation capacity, followed by peptide 6 and L83–S112 C-terminal TGF-β1 and then by full-length TGF-β1; Figure S2: Semi-quantification of Alcian Blue staining on the 14th day of differentiation by measuring the absorbance at 600 nm. Peptide 8 exhibited the highest differentiation capacity, followed by peptide 6 and L83–S112 C-terminal TGF-β1 and then by full-length TGF-β1; Figure S3: Cell-concentration dependent rheological properties of hDPSCs in aMEM at physiological temperature: (a) dynamic viscosity as a function of shear rate, (b) G′ and (c) G″ as a function of time.
