# Supplementary material for: In Vitro Chondrogenesis Induction by Short Peptides of the Carboxy-Terminal Domain of Transforming Growth Factor β1
Source: Biomedicines. 2023 Nov 29;11(12):3182. doi: 10.3390/biomedicines11123182 (PMC10740954; doi:10.3390/biomedicines11123182)
Supplement: Supplementary file 1 [file biomedicines-11-03182-s001.zip › biomedicines-2643255-supplementary.pdf]

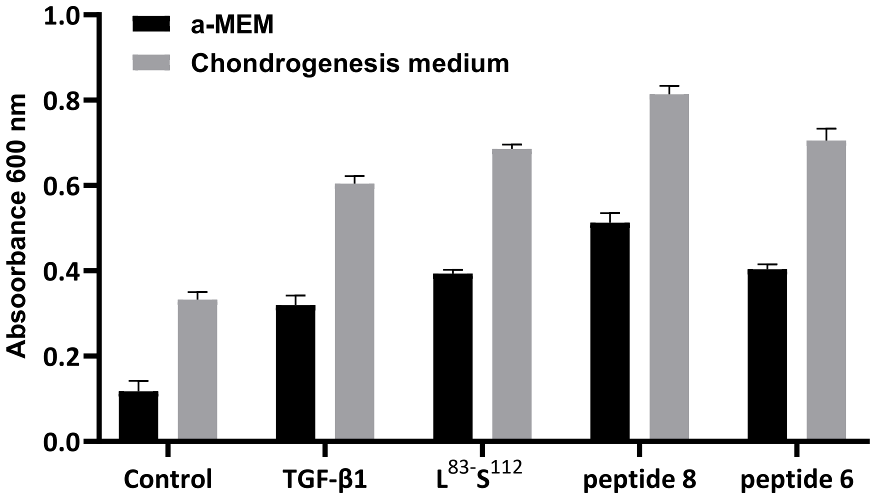

Figure S1. Semi-quantification of Alcian Blue staining on the 14th day of differentiation by measuring the absorbance at 600 nm. Peptide 8 exhibited the highest differentiation capacity, followed by peptide 6 and L^{83}–S^{112} C-terminal TGF-β1 and then by full-length TGF-β1.

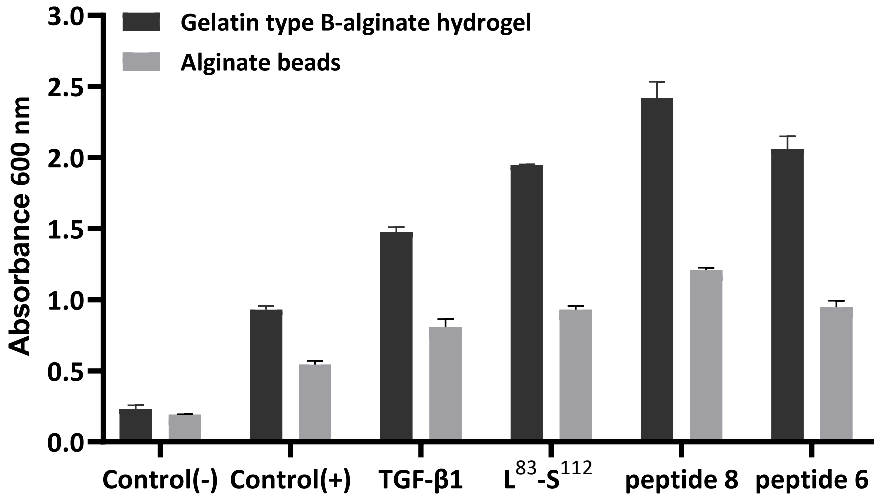

Figure S2. Semi-quantification of Alcian Blue staining on the 14th day of differentiation by measuring the absorbance at 600 nm. Peptide 8 exhibited the highest differentiation capacity, followed by peptide 6 and L^{83}–S^{112} C-terminal TGF-β1 and then by full-length TGF-β1.

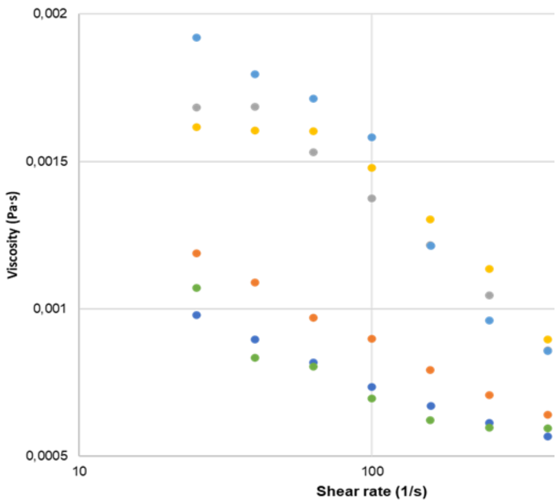

(**a**)

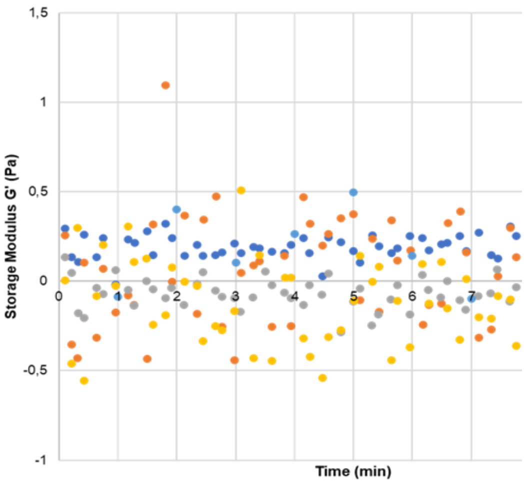

(**b**)

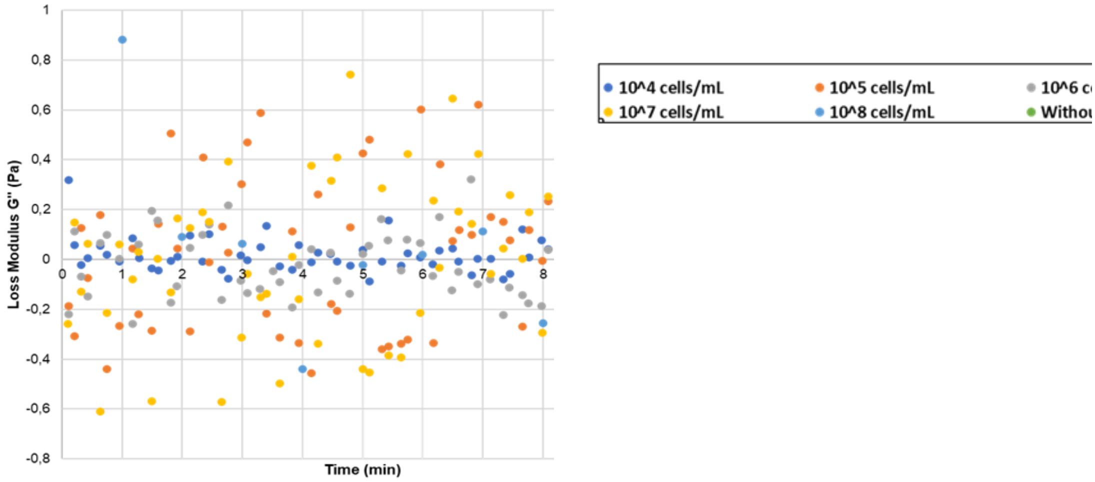

(c)

Figure S3. Cell-concentration dependent rheological properties of hDPSCs in aMEM at physiological temperature: (a) dynamic viscosity as a function of shear rate, (b)G′ and (c) G′′ as a function of time.
